# Serological responses to prednisolone treatment in leprosy reactions: study of TNF-α, antibodies to phenolic glycolipid-1, lipoarabinomanan, ceramide and S100-B

**DOI:** 10.1186/1476-511X-13-119

**Published:** 2014-07-28

**Authors:** Renuka Raju, Sujai Suneetha, Rupendra S Jadhav, MeherVani Chaduvula, Sara Atkinson, Suman Jain, Leo H Visser, Loretta Das, Ravindra Panhalkar, Vidyagouri Shinde, Parphananda P Reddy, Pramila Barkataki, Diana NJ Lockwood, Wim H Van Brakel, Lavanya M Suneetha

**Affiliations:** 1 CODEWEL Nireekshana ACET India, Narayanaguda, Hyderabad 500029 AP, India; 2 Department of Microbiology, Government Institute of Science, Madame Cama Road, Mumbai, India; 3 London School of Hygiene & Tropical Medicine, London, UK; 4 Department of Neurology and Clinical Neurophysiology, St. Elisabeth Hospital, Tilburg, The Netherlands; 5 The Leprosy Mission Hospital, Naini, Allahabad, UP, India; 6 Miraj Civil Hospital, Miraj, Maharashtra, India; 7 Blue Peter Public Health & Research Centre (BPHRC), Hyderabad, AP, India; 8 Mahavir Medical Research Center, 37/165, BhagwanMahavir Marg, AC Guards, Masab Tank, Hyderabad, AP, India; 9 The Leprosy Mission Hospital, Faizabad, UP, India; 10 Leprosy Unit, Royal Tropical Institute, Amsterdam, The Netherlands

**Keywords:** Leprosy, *TNF-α*, Corticosteroids, Prednisolone, PGL-1, S-100B, LAM, Ceramide

## Abstract

**Background:**

Corticosteroids have been extensively used in the treatment of immunological reactions and neuritis in leprosy. The present study evaluates the serological response to steroid treatment in leprosy reactions and neuritis.

**Methods:**

Seven serological markers [TNF-α, antibodies to Phenolic glycolipid-1 (PGL-1 IgM and IgG), Lipoarabinomannan (LAM IgG1 and IgG3), C2-Ceramide and S100 B] were analyzed longitudinally in 72 leprosy patients before, during and after the reaction. At the onset of reaction these patients received a standard course of prednisolone. The levels of the above markers were measured by Enzyme linked immunosorbent assay (ELISA) and compared with the individuals own value in the month prior to the reaction and presented as percentage increase.

**Results:**

One month before the reaction individuals showed a varying increase in the level of different markers such as *TNF-α* (53%) and antibodies to Ceramide (53%), followed by to PGL-1 (51%), S100B (50%) and LAM (26%). The increase was significantly associated with clinical finding of nerve pain, tenderness and new nerve function impairment. After one month prednisolone therapy, there was a fall in the levels [*TNF-α* (60%), C2-Ceramide (54%), S100B (67%), PGL-1(47%) and LAM (52%)] with each marker responding differently to steroid.

**Conclusion:**

Reactions in leprosy are inflammatory processes wherein a rise in set of serological markers can be detected a month before the clinical onset of reaction, some of which remain elevated during their action and steroid treatment induces a variable fall in the levels, and this forms the basis for a variable individual response to steroid therapy.

## Introduction

Leprosy is a chronic infectious disease caused by the bacteria *Mycobacterium leprae*[1]. The clinical course of leprosy is often interrupted by acute episodes of immunological reactions (Type 1 and Type 2 reactions) that trigger inflammatory processes. Reactions often cause damage to peripheral nerves [2]. Corticosteroids, mainly Prednisolone continues to be the mainstay in the management of reactions and nerve damage in leprosy [3]. The mechanism of action of corticosteroids is primarily through the suppression of pro-inflammatory cytokines including tumor necrosis factor-alpha (TNF-α) [4]. Clinical response to steroids is variable [5-7] with only 50 to 80% showing a significant clinical improvement in nerve function. Lockwood et al. have measured TNF-α, interferon gamma (IFN-γ), and interleukin-2 (IL-2) levels in tissues and cells of patients with reversal reaction treated with steroids and found that there was a significant reduction in cytokine levels including TNF-α in most of the patients except a few who continued to maintain elevated levels even after 28 days of treatment [8,9].

A longitudinal study of nerve function impairment in reaction (INFIR study) was carried out in a cohort of leprosy patients to identify potential early markers for reactions and nerve function impairment (NFI) [10]. The markers evaluated were Tumor necrosis alpha (TNF-α), antibodies to mycobacterial Phenolic glycolipid −1 (PGL-1) and Lipoarabinomannan (LAM) and antibodies to cell surface component Ceramide and cytosolic and membrane component S100-B and the dynamics of these markers studied as they have been implicated in the pathogenesis of reactions and nerve damage. Cross sectional analysis carried out on these plasma markers at the time of diagnosis did not show any significant differences in the group of patients with or without reaction [10,11] and hence we designed a novel analysis based on individualistic responses of plasma markers to steroid therapy. The response of these markers to steroid therapy over time was investigated. The present study was carried out in 72 MB patients of this cohort who developed reaction and the samples were analyzed before, during and after the reactional event. The markers were compared with individuals own ‘pre-reaction time’ and ‘post reaction time’ levels and each of the patients were treated with standardized steroid therapy [11]. Inter individual differences in immunological responses is common observation. However, our hypothesis is that individual show variable response to serological markers during steroid treatment. Additionally, we found that there were no reports on the *in vitro* effect of steroids on TNF-α production in short term cell culture in leprosy patients and therefore carried out an *in vitro* study.

In the present study we have evaluated seven serological markers concomitantly before, during and after the reactions in patients treated with steroids.

## Materials and methods

Permission for the INFIR (ILEP Nerve Function Impairment in Reactions) cohort study was obtained from the Indian Council of Medical Research and ethical approval was given by the Research Ethics Committee of the Central JALMA Institute for Leprosy in Agra. Informed consent was obtained from all patients at each center where subjects were recruited.

### Study population

The INFIR cohort comprised of 303 newly registered patients at The Leprosy Mission (TLM) hospitals in Naini and Faizabad, in Uttar Pradesh, India. These patients were followed up for 2 years and serum samples were collected every month in the first year and alternately in the second year. For the present study 72 patients in reactions were selected out of which borderline tuberculoid (BT) were 45 (with bacillary index (BI) 0 to1), borderline lepromatous (BL) were 22 and lepromatous leprosy (LL) were 5 (with BI 1 to 5). All patients were put on WHO multidrug therapy (MDT). A detailed description of the study design has already been published [11,12]. Patients who were clinically diagnosed with Type I and/or nerve function impairment (NFI) were treated with prednisolone according to the standard protocol [12-14] for reactions and neuritis (daily dosage not exceeding 1 mg/kg body weight for 3–6 months). The patients who presented with reactions or recent NFI at recruitment were excluded from the present analysis. A group of 72 patients were identified who developed a reaction (considered an event) and NFI during the course of follow up and formed the focus of this analysis. A separate data sheet was prepared which enabled us to concomitantly evaluate all the plasma markers.

**Figure 1:**
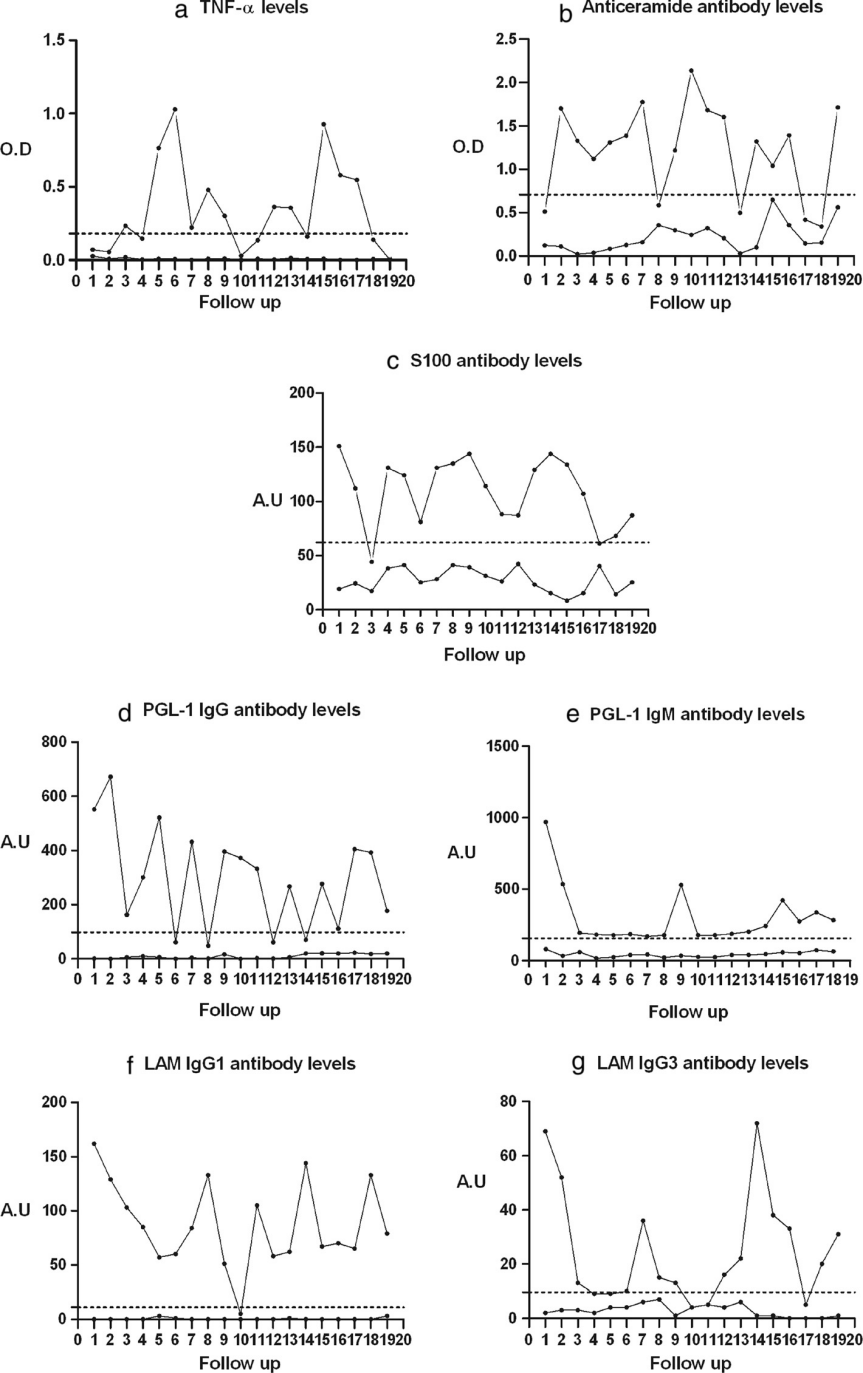
**Response of serological markers to steroids (a to g): Representative 20 month follow up graphs of different individuals showing high or low levels of serological markers such as TNF-α; antibodies to Ceramide; S100; PGL-1 IgG; PGL-1 IgM; LAM IgG1 and LAM IgG3.** The dotted line is the mean level for each marker 1a to 1b in optical density (O.D) at 450 nm and 1c to 1 g in arbitrary units (A.U).

In these 72 patients a sample of serum was available one month prior to the reaction, at the time of reaction and one month after the reaction. The samples were analyzed for seven serological markers PGL-1 (IgM & IgG), LAM (IgG1 & IgG3), Ceramide and S100 antibodies and cytokine *TNF-α* by ELISA. Serological markers were measured by optical density (OD) at 450 nm [(TNF-α & Ceramide) Figure 1a & b] and was converted into arbitrary units [(PGl-1 IgM & IgG and LAM IgG1 and IgG3) Figure 1c to g)] for graphical representation. Individual patient values were compared with the reaction time measure as the percentage increase or decrease of their own levels. This type of analysis helped us to normalize inter-subject variation in the level of markers.

#### ELISA

Antibodies to PGL-1 (IgM & IgG), LAM (IgG1 & IgG3), Ceramide, S100 and cytokine *TNF-α* were measured by ELISA. Antigens were to be tested were originally dissolved in suitable solvent like de-ionized water (S-100 and PGL-1), or 70% methanol in PBS (ManLAM) or chloroform: methanol (3:1) and further dilution was carried out in absolute alcohol (0.5 mg/ml) in PBS (ceramide). ELISAs were carried out in 96 well plates (Immulon & Dynatech) coated with the antigen at a concentration of 0.1 mg/well in 0.05 M carbonate-bicarbonate buffer pH 9.6 by incubating overnight at 37°C (for S-100, PGL-I and LAM). For anti-ceramide, the antigen was further diluted in absolute alcohol then suspended in PBS and sonicated immediately prior to coating to obtain uniform suspension. Optical density (OD) of all the markers was measured at 450 nm. The details of ELISA and methodology have been presented in an earlier publication [10].

Additionally, ***TNF-α*** production in cultured lymphocytes was measured in treated leprosy patients (n = 13) and healthy subjects (n = 11).

### PBMC cell culture

Peripheral blood mononuclear cultures were set up with isolated lymphocytes from leprosy [15,16]. In brief, 5 ml of heparinized venous blood was collected from 13 treated leprosy patients and 11 healthy subjects. Equal numbers of cells of each sample were taken for the stimulation assay. 2 × 10^6^ cells/well were used for culture [17]. Stimulation of cells was carried out by phytohemagglutinin [(PHA) (5 μg/ml)], Concanavalin A [(Con A) (10 μg/ml)] and methyl prednisolone (5 μg/ml) [(Sigma, St. Louis, USA; Pharmacia and Upjohn company, Michigan, USA)]. Cultures were incubated at 37°C in a 5% CO_2_ incubator. Cells were cultured for 24 hr and the supernatant was stored at −70°C until the TNF-α ELISA (R & D Systems, UK) was carried out. TNF-αlevel in un-stimulated, PHA and Con A and methyl prednisolone treated cells were arbitrarily taken as 100% in both leprosy and healthy subjects.

### Statistical analysis

The results of ELSIA & *in vitro* levels of TNF-α were expressed as percentages and mean ± SD respectively and the data were analyzed statistically by the ANOVA one-way analysis of variance (F) using GraphPad Prism version 5.

## Results

### Serological markers during the follow up

Individuals expressed high or low levels of serological markers during the 2 year follow up. Figure 1a-g shows individuals representing high and low levels of each marker TNF-α, antibodies to Ceramide, S100, PGL-1(IgM & IgG) and LAM (IgG1 & IgG3). Variable periodicity of peak occurrence of serological markers could be observed in individuals.

### Percentage change in levels of serological markers before, during and after reaction

Seventy two patient samples were analyzed for all the seven markers mentioned earlier. The analysis was carried out at three time points i.e., before, during and after the reaction. Prednisolone was initiated at the time of reaction. At the time point of the reaction all seven markers levels were considered 100% (arbitrary) to which before and after the reactional levels were compared. Table 1 shows the increase or decrease of the seven markers before and after reaction. Out of seven markers before reaction the levels of TNF-α and Ceramide showed maximum increase of 53% and LAM showed minimal increase of 36%.

**Table 1:** Individuals showing increase and decrease of serological markers before & after reaction

**S. no**	**Serological markers (n = 72)**	**Before the event increase**	**After the event decrease**	**Range of inhibition (%)**
1.	Ceramide	38 (53%)	39 (54%)	6.4 to 99.5
2.	S100	36 (50%)	48 (67%)	1.7 to 100
3.	PGL IgG	37 (51%)	35 (49%)	6.1 to 89
4.	PGL IgM	35 (49%)	32 (44%)	5.3 to 86
5.	LAM IgG1	23 (32%)	37 (51%)	22.2 to 96.6
6.	LAM IgG3	29 (40%)	38 (53%)	5.3 to 100
7.	TNF-α	38 (53%)	43 (60%)	9.4 to 99

Individuals showing increase a month before the reaction and decrease a month after the reaction and the percentage inhibition (in parentheses) profile of each serological marker with the prednisolone treatment*.*

**Figure 2:**
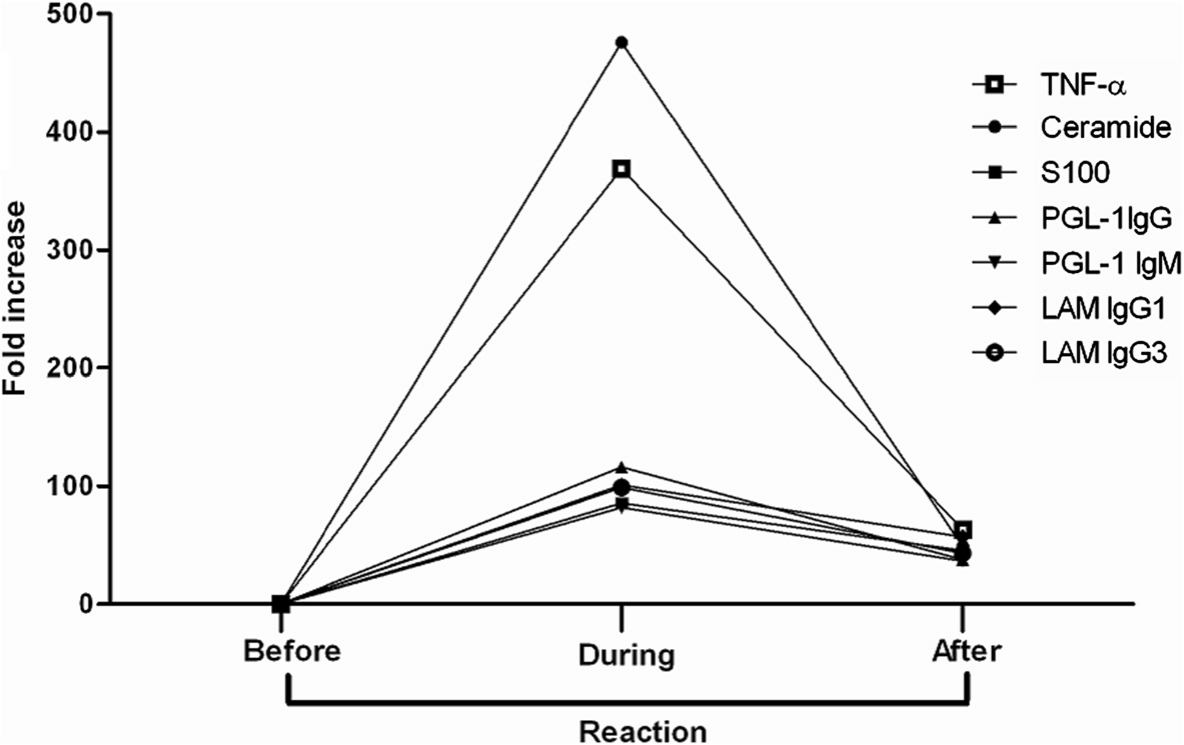
**Effect of steroid therapy on seven serological markers.** Percentage increase or decrease of seven serological markers (TNF-α*,* antibodies to Ceramide; S100; PGL-1 IgG; PGL-1 IgM; LAM IgG1 and LAM IgG3) in leprosy patients before, during and after the reaction and their association with steroid therapy*.*

The percentage of individuals showing an increase in the level of markers one month prior to reaction was in the order of - 53% *TNF-α* and 53% Ceramide antibody, 51% PGL IgG antibody, 49% PGL IgM antibody, 50% S100 antibody, 40% LAM IgG3 antibody and 32% LAM IgG1 antibody (Table 1).Fold change in the levels of the seven serological markers before, during and after reaction were shown in Figure 2. We designed a comparison with the value of each molecule one month prior to the reaction as the baseline measure. Then to normalize intra subject variations, percentage increase and decrease (after steroid therapy) to the baseline measure was derived.

Quantitative increase in levels of all seven markers a month prior to reaction was found only in a single individual and all were decreased a month after reaction with steroid therapy in only two individuals. All the other individuals showed an increase of any of the seven markers in a combination of 2 to 6 markers before reaction. When any one of the seven tests were carried out on an individual patient an increase level of markers before the reaction was observed only in 7% whereas when any of two or more tests (out of 7 tests) were carried out there was an increase to 89% (Table 2).

**Table 2:** Serological markers and their association with nerve pain, tenderness and NFI

**S. no**	**No of serological markers**	**% increase in serological markers before reaction (n = 72)**	**% decrease in serological markers after reaction (n = 72)**	**During the reaction percentage increase**		
				**Nerve pain**	**Nerve tenderness**	**New Nerve Function Impairment (NFI)**
1.	One	7	4.2	20	0	80
2.	Two	15.3	14	36.4	9	72.7
3.	Three	31	25	31.8	27.2	54.5
4.	Four	22.2	26.4	62.5	31.2	43.7
5.	Five	14	21	20	20	60
6.	Six	6	7	25	25	25
7.	Seven	1.4	3	0	0	100

Any of the serological markers TNF-α, antibodies to Ceramide; S100; PGL-1 IgG; PGL-1 IgM; LAM IgG1 and LAM IgG3 increased one month before the reaction and decreased a month after the reaction and their association with clinical symptoms of nerve pain, nerve tenderness and new nerve function impairment.

When the same analysis was done on the subjects a month after the reaction with any single test only 4.2% showed a decrease; however any of two or more tests in combination showed a decrease of 93.4%. Maximum inhibition was observed in S100 antibody (67%) followed by TNF-α (60%). The range of inhibition for each of the seven serological markers varied from individual to individual (Table 1).

### Association of serological marker levels at time of reaction with nerve damage

Of the patients, 53.2% showed an increase of any three or four serological markers before the reaction showed clinically either individually or in combination an increase of nerve pain, nerve tenderness and new NFI at the time of reaction (Table 2)*.*

### *In-vitro* effect of methylprednisolone on TNF-α release

PBMCs derived from leprosy and healthy subjects were stimulated and cultured in the presence of PHA, Con A and methylprednisolone. The levels of TNF-αin un-stimulated media in *healthy and* leprosy are 40 ± 18 pg/ml (0.23 ± 0.11) and 16 ± 7 pg/ml (0.09 ± 0.05) respectively (Table 3). The extent of TNF-α inhibition in healthy subjects for methylprednisolone was 53.1% and in leprosy affected was 65.3%. There was no significant difference in the inhibition patterns of TNF-αlevel between healthy subjects and leprosy.

**Table 3:** ***In vitro *** **TNF-α levels in stimulated peripheral blood mononuclear cells (PBMC)**

**S. no**	**Description**	**Healthy subjects (n = 11)**	**Leprosy patients (n = 13)**
1	Unstimulated Media	0.23 ± 0.11	0.09 ± 0.05
2	PHA + conA	0.81 ± 0.66	1.08 ± 0.56
3	Methyl prednisolone (DC)	0.42 ± 0.46	0.22 ± 0.08

In vitro TNF-α levels in unstimulated media, PHA + conA stimulated and methyl prednisolone (DC) was expressed as optical density (OD) as mean ± SD.

## Discussion

It is estimated that 2.1 million people around the world have deformities due to leprosy. Reactions and nerve damage in leprosy require prompt and adequate treatment with steroids/anti-inflammatory drugs failing which permanent deformities result [18,19]. Each of the markers mentioned earlier have been found to play a role in the pathogenesis of reactions and nerve damage in leprosy by other groups [10,19,20]. However each of these have not been studied individually and together in a single individual with values before, during and after a reaction.

We hypothesized that individuals respond differentially to steroids and that is reflected in increase or decrease of the levels of these serological markers and this variability is associated with reactions and nerve damage.

The follow up data of patients with leprosy reactions showed (Figure 1a to g) spikes at periodic intervals suggesting simmering inflammation in leprosy. The periodic increase and decrease in the markers could be due to the release of processed mycobacterial antigen into the immunological milieu and the concomitant rise of inflammatory markers. Similar phenomenon was observed in other progressive neurodegenerative diseases such as Multiple Sclerosis [21].

Of the individuals, 47% showed an increase in the level of markers when compared to their own individual existing level before the reaction. This is a unique analysis comparing the individual’s level of each molecule to their existing level prior and after reaction. The individual variability in the level of expression of markers were normalized and presented as percentage increase or decrease. This finding established that leprosy patients show a variable increase of different markers before reaction. Our analysis showed two or four serological markers when tested in combination showed about 70% sensitivity to detect reaction. The maximum fold increase was observed in Ceramide antibody followed by TNF-α, followed by S100 antibody and PGL-1 (Table 1 and Figure 2) suggesting the combination of these four serological markers could be a choice to understand reaction and their relationship to nerve damage. In addition, to the above mentioned four serological markers other inflammatory and autoimmune markers which have a fold increase such as Interferon-inducible protein-10 (IP-10) [22] and Myelin P0 [23-25] should be explored for understanding the nerve damage.

As shown in Table 1 nerve pain, tenderness, new NFI was considered at the time of reaction and were correlated with increase in any of the serological markers individually or in combination. When all seven markers showed an increase during reaction it was associated with new nerve function impairment. The markers were not significantly different a month after the reaction. The major limitation or constraint in leprosy studies is defining the onset of infection and the exact time point of reaction and nerve damage which creates a difficulty in the enrollment of homogenous patient group. Further studies would help in understanding the association between clinical symptoms (nerve pain, nerve tenderness and new NFI) and serological markers during progression of nerve damage.

We studied the steroid response after the reaction and our analysis showed a cumulative decrease in the levels of the markers about 65.4% (when four serological markers were tested at a time). The maximum decrease was observed in antibodies to S100, TNF-α and antibodies to Ceramide & LAM (Table 1). A study on steroids treatment of reaction and changes in the inflammatory cytokines showed that TNF-αand other cytokines continue to be produced for a considerable time during and after the reaction [17] suggesting that a sustained inhibition of the inflammatory process is warranted in the management of reactions. As clinical reactions are known to precede nerve damage, inhibition of S100 antibody and TNF-α in reaction could be a molecular mechanism by which reactions are controlled, thus facilitating a quick recovery of nerve function in leprosy.

Steroids have been the mainstay of treatment of reactions and prevention of nerve impairment. Even though the number of samples assayed by *in vitro* were small, PBMC when stimulated with PHA/Con A showed a significant increase in TNF-αproduction in patients as compared to healthy subjects (Table 3). Inhibition of TNF-α by steroid (DC) was 65.3% by *in vitro* as compared to 60% *in vivo* (Table 1) suggesting partial response in leprosy reactions. The present study has shown that steroids do not produce a consistent and/or sustained suppression of all the markers associated with reaction and nerve damage. There is a need for alternate drug combinations to manage reaction and prevent nerve damage. Azathioprine and thalidomide are now being used as substitutes for steroids [26-28]. Considering the serological & *in vitro* results this study recommends a combination of steroids and other drugs that can effectively prevent leprosy nerve damage.

In conclusion, this study helps in understanding the responses of a leprosy individual in reaction to steroids *in vivo* and *in vitro*. There is a heterogeneity in the immunological responses to steroids and suggest that the future therapies should be multi component and individually tailored. Inhibition of autoantibodies with steroids could be a significant mechanism in preventing nerve damage. An increase in the serological markers before a reaction had an association with clinical symptoms and signs of nerve pain, tenderness, and new NFI and thus needs to be considered in the management of nerve damage. Identification of steroid responders and non-responders by an *in vitro* test could benefit the physician in better management of reactions and nerve damage.

Future studies on protein microarray would provide a comprehensive evaluation of other inflammatory and regulatory markers that are involved in leprosy reaction and that are down regulated in response of prednisolone therapy. Furthermore it would be interesting to study whether similar immunological and molecular mechanisms occur in Type II reactions of leprosy and the role of steroids treatment in nerve damage.

## Competing interests

The authors declare that they have no competing interests.

## Authors’ contribution

RR, SS, MC, LMS wrote the manuscript. RSJ designed PGL, LAM and S100B experiments. RK and VS conducted PGL, LAM and S100B experiments. RR, CM, LMS designed and conducted TNF-α and Ceramide experiments. SA designed *in vitro* steroid assay. RR, LMS, MC, RSJ, RK, VS helped in the analysis of the data. SS, LD, SJ, PB, DL are the clinicians involved in the study. LV & PPR was involved in the editing of MS. WVB is the project coordinator of INFIR. All authors read and approved the final manuscript.
